# University of Pennsylvania

**Published:** 1844-04-06

**Authors:** W. E. Horner

**Affiliations:** Dean of the Medical Faculty


### UNIVERSITY OF PENNSYLVANIA.

### MEDICAL DEPARTMENT.

At a Public Commencement, held on Thursday, April 4th, 1844, in the Musical Fund Hall, Locust street, the Degree of Doctor of Medicine was conferred by the Rev. John Ludlow, Provost, upon the following gentlemen; after which an Address was delivered by Dr. Horner, Professor of Anatomy.

| | Subject of Theses. |
|---|---|
| | *New Hampshire.* |
| Thomas E. Hatch, | Apoplexy. |
| | *Vermont.* |
| Charles W. Gleason, | Capillary circulation. |
| | *Massachusetts.* |
| William W. Bliss, | Delirium Tremens. |
| William F. Channing, | Application of Chemistry to Physiology. |
| Benjamin Heywood, | Spermatorrhea. |
| Henry Osgood Stone | Polypus of the nose. |
| | *Rhode Island.* |
| Edmund V. Hathaway | Scarlatina. |
| | *New York.* |
| Wm. C. Benedict | Erysipelas with black tongue. |
| Joel B. Phinney | Cutaneous diseases. |
| Leander B. Phinney | Ulcer of the cornea. |
| | *New Jersey.* |
| John W. Blackfan | Scarlatina. |
| Leison E. English | Influenza. |
| Ephraim Holmes, Jr. | Acute Peritonitis. |
| Eugene Jobs | Diabetes. |
| Joseph Parrish | Scrofulous diathesis. |
| Wm. R. Phillips | Electricity. |
| | *Pennsylvania.* |
| Almon Z. Berdin | Intermittent Fever. |
| Adam S. Bare | Disease of the ovaries. |
| Samuel R. Bleight | Intermittent Fever. |
| Martin H. Boyel | Structure of the nervous system. |
| Thomas F. Cullen, | Anchylosis of knee-joint. |
| William Curran, | Dyspepsia. |
| John Curwen | Scrofulous Ophthalmia. |
| Robert Davidson | Acute Dysentery. |
| Ebenezer V. Dickey | Erysipelas. |
| Samuel Dilworth, Jr. | Influence of mind upon the body. |
| George Dock, | The blood as modified by disease. |
| Abraham H. Fetherman | Pertussis. |
| Charles S. Gauntt | Mercury and its compounds. |
| Lewers D. Gray | Purpose and province of Medical science. |
| Alfred W. Green | Bronchocele. |
| Theodore B. Hale | Dysentery. |
| Edward A. Hall | Remittent Fever, |
| Andrew B. Happer | Influence of Medicine. |
| Robert P. Harris | Veratria. |
| Bernard Henry | Aneurism. |
| William V. Keating | Typhoid Fever. |

| | Subject of Theses. |
|---|---|
| William L. Latta | Whooping cough. |
| Joseph Leidy | Comparative anatomy of the eye. |
| David B. Marshall | Variola. |
| Edward R. Mayer | Nitrate of silver. |
| John H. B. McClellan | Medullary Fungus. |
| William S. McIlhenny | Dew. |
| Joseph Priestly | Wounds of Arteries. |
| Lewis Royer | Delivery of Placenta. |
| Samuel Sandt | Dyspepsia. |
| Samuel D. Scott | Physiology of the eye-ball. |
| Washington Sherman | Feigning of Disease. |
| Francis Sims | Rheumatism. |
| John W. Snowden | Diagnostic sign of Uterine Pregnancy. |
| Moreton Stille | Pathology of cyanosis. |
| Edward S. Wilcox | Fractures. |
| Samuel Wilson | Pleuritis. |
| Wm. Wilson | Use of tartar emetic in obstetrical practice. |
| Thomas H. Wilson | Dysentery. |
| Thomas Wood | Inversion of the womb. |

*Delaware.*

| | |
|---|---|
| John T. M. Cardeza | Hepatic Abscess. |
| Julian Rogers | Acute Hepatitis. |

*Maryland.*

| | |
|---|---|
| Wm. Tyler, Jr. | Scarlatina. |
| Richard K. Osbourn | Medical Treatment of Stuangulated Hernia. |
| Wm. Gray Palmer | Menstruation. |

*Virginia.*

| | |
|---|---|
| Junius L. Archer | Puerperal Fever. |
| Peter Barr | Jaundice. |
| John T. Basye | Croup. |
| John Roy Baylor | Intermittent Fever. |
| James W. Bryant | Scarlatina, |
| Edwin Gray Buckles | Acute Meningitis. |
| John M. Burton | Dysentery. |
| Lewis W. Carter | Diarrhœa. |
| James L. Clarke | Phenomena of catamenia. |
| Wm. J. Clark | Regimen for dyspeptics. |
| Thomas J. Cooke | Atmospheric air. |
| Octavius A. Crenshaw | Spermatorrhea. |
| Richard Dillard | Strictures of Urethra. |
| Wm. T. Eldridge | Fractures. |
| James T. Foley | Cynanche Trachealis. |
| John N. Garnett | Chronic Gastritis. |
| Robert Graves | Fracture of the femur. |
| John B. Green | Icterus. |
| Solomon Green | Dyspepsia. |
| John P. Hale | Diseases of Menstruation. |
| James W. Hopkins | Cæsarean section. |
| Etheldred W. Lundy | Cynanche Trachealis. |
| Randolph F. Mason | Scarlatina. |
| George W. O. Maupin | Scarlatina. |
| A. Russell Meem | Calomel, use of, in febrile affections. |
| George B. Moffett | Importance of the knowledge of physiology and diagnosis in the treatment of disease. |
| Edgar Moss | Amaurosis. |
| James A. Reid | Belladonna. |
| Angus A. Rucker | Iodine. |
| Granville Smith | Aneurism. |
| Lewis T. Taliaferro | De febre biliosa remittente. |
| John B. Taylor | Intestinal Worms. |
| George F. Terrill | Menispermum Canadense. |
| John Wickham | Inflammation of the kidneys. |
| Benjamin Wilson | Bilious Remittent Fever. |
| Wm. M. Wilson | Scarlatina. |
| J. Lewis Woodville | Hernia. |

*North Carolina.*

| | Subjects of Theses. |
|---|---|
| Kemp P. Alston | Laryngitis Membranacea. |
| Richard P. Ashe | Cataract. |
| John P. Clingman | Fever. |
| Peter Custis | Acute Gastritis. |
| George S. Dejarnatte | Mental influence over the organic functions. |
| Henry W. Faison | Remittent Fever. |
| Peter S. Foster | Koinomiasmata. |
| Chauncey W. Graham | Pertussis. |
| Wm. H. Hughes | Puerperal Peritonitis. |
| Henry Joiner | Intermittent Fever. |
| Wm. L. Long | Remedial and poisoning influence of lead. |
| Murdock M'Leod | Acute Pleuritis. |
| Rawley A. Scales | Puerperal Fever. |
| Elias F. Shaw | Intermittent Fever. |
| Joseph J. Summerell | Influence of opium in autumnal fevers. |
| Moses B. Taylor | The pulse and its modifications. |
| Elias A. White | Gastritis. |
| Henry F. Williams | Menstruation. |

*South Carolina.*

| | |
|---|---|
| Joseph S. Crane | Inflammation. |
| Edward F. Gregg | Death. |
| Benjamin S. Screven | Circulation. |
| John,G. Seabrook | Neuralgia. |
| Wm. Wylie | Epidemic. |

*Kentucky.*

| | |
|---|---|
| Addison Dimmitt | Physiology of the circulation. |
| Wm. H. Mackie | Poisoned Wounds. |
| Alexauder K. Marshall | Superfœtation. |
| Wm. W. Watson | Tobacco. |

*Georgia.*

| | |
|---|---|
| James R. M. Reid | Remittent Fever. |
| John H. Roberts, Jr. | Acute Gastritis. |

*Mississippi.*

| | |
|---|---|
| Mills T. Anderson | Counter-Irritation. |
| Felix Gorman | Intermittent Fever. |
| David D. Irwin | Yellow Fever. |
| Wm. S. M'lutosh | Chronic Hepatitis. |

*Tennessee.*

| | |
|---|---|
| Franklin M. Compton | Inflammation. |
| Robert J. Farquharson | Phenomena and causes of inflammation. |
| Junius H. Howell | Poisoning by opium. |
| James E. Knott | Protochloride of mercury. |
| James H. Sharp | Leucorrhœa. |

*Ohio.*

| | |
|---|---|
| John A. Pettit | Rubeola Vulgaris. |
| Clement H. Wesson | Primary Syphilis. |

*Alabama.*

| | |
|---|---|
| Edward Anderson | Atmospheric air. |
| James W. Harris | Menorrhagia. |
| Joseph Hearne | Functions of the brain. |
| J. Claxton Hughes | Puerperal Fever. |
| James J. Mundy | Congestive Fever. |
| John B. Sykes | Heat as a morbific agent. |
| T. Wilkinson | Moral Medicine. |

*Louisiana.*

| | |
|---|---|
| Charles A. Hardey | Congestive Fever. |
| Daniel C. Holliday | The eye a diagnostic sign of disease. |
| Edward L. Skillman | Variola. |

*Florida.*

| | |
|---|---|
| Wm. Henry Joyner | Acute Peritonitis. |

At the Commencement in July, 1843, the Degree of Doctor of Medicine was conferred upon the following gentlemen:

*Virginia.*

| | |
|---|---|
| Anderson Bagley | Fractures of Thigh. |
| Theordore A. Michie | Acute Rheumatism. |

*North Carolina.*

| | |
|---|---|
| James H. Boon | Fractures. |

Total for Collegiate Year, 153.

W. E. HORNER, M. D.,
*Dean of the Medical Faculty.*

